# Long noncoding RNAs in hematopoiesis

**DOI:** 10.12688/f1000research.8349.1

**Published:** 2016-07-20

**Authors:** Xu Zhang, Wenqian Hu

**Affiliations:** 1 Department of Biochemistry and Molecular Biology, Mayo Clinic, Rochester, MN, 55905, USA

**Keywords:** hematopoiesis, noncoding RNAs, gene expression

## Abstract

Mammalian development is under tight control to ensure precise gene expression. Recent studies reveal a new layer of regulation of gene expression mediated by long noncoding RNAs. These transcripts are longer than 200nt that do not have functional protein coding capacity. Interestingly, many of these long noncoding RNAs are expressed with high specificity in different types of cells, tissues, and developmental stages in mammals, suggesting that they may have functional roles in diverse biological processes. Here, we summarize recent findings of long noncoding RNAs in hematopoiesis, which is one of the best-characterized mammalian cell differentiation processes. Then we provide our own perspectives on future studies of long noncoding RNAs in this field.

## Introduction

One of the exciting findings from genomic studies over the past decade is the identification of a large number of long noncoding RNAs (lncRNAs) in mammalian transcriptomes. These transcripts are longer than 200 nucleotides in length, and though structurally similar to mRNAs, lncRNAs do not have functional protein-coding capacity. In addition, lncRNAs are usually expressed at lower levels compared with mRNAs, and most lncRNAs are not evolutionarily conserved in primary sequences ^1– 6^. Interestingly, transcriptomic studies revealed that many lncRNAs are expressed in a manner highly specific to cell types, tissues, developmental stages, and pathological conditions in mammals ^1, 2^, suggesting that these transcripts are involved in these biological processes. Consistent with this, the biological functions of many lncRNAs are being characterized in diverse biological and pathological settings ^7– 9^. Mechanistically quite different from small RNAs, such as microRNAs that control gene expression predominantly at one particular step (mRNA translation and degradation), lncRNAs can regulate gene expression at multiple levels, from transcription and splicing to mRNA translation and degradation, in mammals. There are several outstanding reviews summarizing our current understandings of the mechanistic aspects of lncRNA-mediated regulation of gene expression ^10– 14^ and the hematopoiesis-related lncRNAs ^15, 16^. In this review, we focus on biological functions of lncRNAs in hematopoiesis.

For three reasons, the hematopoietic system is one of the best paradigms for studying cellular lineage determination and differentiation in mammals ^17^. First, hematopoiesis is well characterized at the cellular level in both humans and mice, and a single hematopoietic stem cell (HSC) can re-constitute all the cells in the hematopoietic system ^18^. Based on this, many lineage specification and differentiation processes of HSCs and their progenies are characterized. Second, almost all the multi-potential and lineage-determined progenitor cells in hematopoiesis can be enriched or isolated in an almost pure form by flow cytometry using combinations of cell surface markers ^19, 20^. This makes the phenotypic studies of a specific cellular lineage relatively easy. Third, both *in vitro* and *in vivo* assays are well established for functional characterization of hematopoietic progenitor cells ^19^. Collectively, these features of the hematopoietic system greatly facilitate the exploration of both proteins and RNAs controlling cell fate determination and differentiation ^18^.

Here, we summarize recent studies on the biological functions of lncRNAs in hematopoiesis. Particularly, we focus on lncRNAs in the regulation of HSCs and differentiation of several major hematopoietic cell lineages ( Table 1). Then we provide our own perspectives on future studies of lncRNAs in hematopoiesis.

**Table 1.** Examples of long noncoding RNAs in hematopoiesis.

Long noncoding RNA	Cell types	Assay	Observed phenotype	Reference
H19	HSC	Deletion of the upstream region of H19	Reduced adult HSC quiescence and compromise of HSC function	21
LncHSC-1/2	HSC	shRNA knockdown	Impacted HSC self-renewal and differentiation	23
lincRNA-EPS	Erythroid progenitors	shRNA knockdown and overexpression	Essential for maturation of red blood cells	26
alncRNA-EC7	Erythroid	shRNA knockdown	Depletion of BAND 3	24
HOTAIRM1	Myeloid progenitors	shRNA knockdown	Attenuates the maturation of granulocytes	29, 31
Eosinophil granule ontogeny	Eosinophils	shRNA knockdown	Compromises the expression of several proteins that are important for eosinophil development	32
lnc-DC	DCs	RNA interference and overexpression	Vital for DC differentiation in both humans and mice	42
linc-MAF-4	Th1 cell	siRNA-mediated knockdown	Increases the expression of MAF and skews T-cell differentiation toward the Th2 phenotype	40

DC, dendritic cell; HOTAIRM1, HOX antisense intergenic RNA myeloid 1; HSC, hematopoietic stem cell; shRNA, short hairpin RNA; siRNA, small interfering RNA.

## Long noncoding RNAs in hematopoietic stem cell maintenance and differentiation

HSCs are the best-characterized somatic stem cells in mammals. Recent observations suggest that lncRNAs play regulatory roles in HSC biology. For example, epigenetic controlling allele-specific expression of H19, an lncRNA involved in imprinting, is critical for maintaining “stemness” of HSCs. Specifically, using a mouse model with maternal-specific deletion of the differentially methylated region upstream of H19, a critical cis-element in controlling imprinting, the Li group found that the expression of H19 target Igf2 is de-repressed, which results in reduced adult HSC quiescence and compromise of HSC function ^21^. Although in this mouse model the H19 locus is not deleted, removing the critical cis-element inhibits the expression of H19. Interestingly, H19 can also give rise to a microRNA named miR-675, which also represses the expression of the insulin-like growth factor 1 receptor (Igf1r) ^22^. Thus, inhibiting H19 expression may have pleiotropic effects on mouse development. Nonetheless, the results from this genetically modified mouse indicate that proper epigenetic modifications of the lncRNA H19 locus are important for maintaining HSCs. To comprehensively identify and annotate lncRNAs specifically expressed in HSCs, the Goodell lab performed comprehensive transcriptomic profiling on highly purified mouse HSCs followed by a series of stringent filtering steps to exclude the protein-coding genes and the lowly expressed transcripts ^23^. By comparing the transcriptome of HSCs with those of lineage-committed hematopoietic cells, they identified 159 lncRNAs that are enriched in HSCs. Interestingly, short hairpin RNA (shRNA) knockdown of two of these HSC-enriched lncRNAs compromises HSC self-renewal and lineage commitment. Although shRNA-mediated approaches may have potential off-target effects, the results from this study argue that some of these HSC-enriched lncRNAs are functionally important for HSC biology. This study provides a solid foundation for future functional and mechanistic characterization of lncRNAs in HSCs.

## Long noncoding RNAs in erythropoiesis and megakaryopoeissis

Erythroid cells and megakaryocytes are thought to derive from a common progenitor named megakaryocyte-erythroid progenitor (MEP). Using mouse fetal liver erythroblasts, two groups independently performed RNA sequencing (RNA-seq) analysis on erythroid cells at different developmental stages ^24, 25^. Computational analysis on these transcriptomic datasets revealed that about 650 lncRNAs are dynamically expressed during erythropoiesis. Interestingly, by using shRNA-mediated loss-of-function studies on lncRNAs that are significantly induced (more than threefold) during terminal erythroid differentiation, both of these groups show that several erythroid-specific lncRNAs have regulatory roles in generating enucleated reticulocytes, at least *in vitro*, arguing that these noncoding transcripts are new regulators in erythropoiesis. For example, LincRNA-EPS, an lncRNA specific to terminal differentiating erythroblasts, prevents cellular apoptosis during terminal erythroid differentiation by inhibiting the expression of Pycard, a pro-apoptotic gene ^26^. In addition, by integrating the transcriptomic datasets with the chromatin immunoprecipitation-sequencing datasets, they found that many erythroid-specific lncRNAs are regulated by Gata1 and Tal1, two erythroid-important transcription factors. These observations collectively indicate that lncRNAs are targets of transcriptional regulatory networks in erythroid cell differentiation.

In addition to surveying mouse erythropoiesis, several groups surveyed the transcriptomic dynamics in human erythroid cell differentiation by RNA-seq ^25, 27, 28^. Interestingly, comparing lncRNAs expressed in mouse erythroid cells with those from human erythroid cells, Paralkar *et al*. observed that for most lncRNAs, including those that are functionally involved in mouse erythropoiesis, they are poorly conserved in both primary sequence and expression pattern between mouse and human ^25^. This observation suggests that lncRNAs are under different evolutionary pressures compared with that of protein-coding genes.

Paralkar *et al*. also performed RNA-seq analysis in mouse megakaryocytes and MEP cells. Computational analysis on transcriptomic datasets from erythroid cells, megakaryocytes, and MEPs indicates that many lncRNAs are highly specific to one cell type versus the other two. Thus, it would be very interesting to identify and characterize megakaryocyte-specific lncRNAs, as these noncoding transcripts may potentially regulate megakaryopoiesis. Similarly, analyzing the expression patterns of those MEP-specific lncRNAs during differentiation and loss-of-function and gain-of-function studies of these lncRNAs may reveal how these transcripts modulate the biology of the MEP bi-potential progenitor cells, such as self-renewal and lineage specification.

## Long noncoding RNAs in myelopoiesis and innate immunity

The myeloid leukocytes, including eosinophilic granulocytes, basophilic granulocytes, neutrophilic granulocytes, and monocytes, are thought to derive from a common progenitor named granulocyte-macrophage progenitor in the hematopoietic system. Several observations indicate that lncRNAs are involved in modulating myelopoiesis. For example, a human myeloid lineage-specific lncRNA, HOX antisense intergenic RNA myeloid 1 (HOTAIRM1), is upregulated during granulocytic differentiation of myeloid progenitors ^29^. The genomic locus of this lncRNA is located within the HOXA loci, which encode several HOXA transcription factors that are important for myelopoiesis ^30^. Interestingly, shRNA knockdown of this lncRNA compromises the activation of HOXA1 and HOXA4 during myeloid differentiation and attenuates the maturation of granulocytes as determined by cell surface markers ^29^. HOTAIRM1 knockdown is further shown to influence cell cycle arrest at the G1/S transition to inhibit granulocytic maturation in NB4 cells ^31^. This observation strongly suggests that lncRNA-mediated regulation of HOXA gene expression is important during granulocyte differentiation. LncRNAs are also implicated in regulating eosinophil formation during myelopoiesis ^32^. An lncRNA, with primary sequence conserved among human, mouse, and chicken, named eosinophil granule ontogeny (EGO) was identified from the inositol triphosphate receptor type 1 gene locus. This lncRNA is highly expressed in mature eosinophils, and biochemical experiments indicate that this transcript is not associated with ribosomes and does not have conserved open reading frames (ORFs), strongly arguing that it is noncoding. Interestingly, shRNA-mediated loss-of-function studies revealed that reduction of EGO level compromises the expression of several proteins that are important for eosinophil development, suggesting the functional importance of EGO in eosinophilopoiesis. It would be very interesting to explore how this conserved lncRNA regulates gene expression.

The terminal differentiated effector cells, such as macrophages, from myelopoiesis play important roles in innate immunity. Interestingly, several groups observed that many lncRNAs are differentially expressed when macrophages are challenged by bacteria infection ^33, 34^. The Fitzgerald group functionally characterized one such lncRNA named lincRNA-Cox2 ^34^. This lncRNA is upregulated more than 20 fold by the Toll-like receptor signaling pathway in mouse bone marrow-derived macrophages. Importantly, biochemical analysis indicates that linc-RNA-Cox2 is not associated with ribosomes. Detailed functional and molecular mechanistic studies revealed that lincRNA-Cox2 interacts with heterogeneous nuclear ribonucleoproteins A/B and A2/B2 to modulate the expression of several immune response genes during inflammatory signaling.

## Long noncoding RNAs in lymphoid lineages

Lymphopoiesis is the regulated generation of lymphoid cells, including T cells, B cells, natural killer cells, and dendritic cells (DCs) in the hematopoietic system. Transcriptomic studies from numerous groups identified a large number of lymphoid-specific lncRNAs that are differentially expressed during the maturation of these lymphoid cells ^35– 39^. For example, during CD8 ^+^ lymphocyte differentiation, hundreds of lymphoid-specific lncRNAs were identified ^35^. More recently, comprehensive transcriptomic surveillances in human lymphoid cells identified over 3,000 lncRNAs, and many of these transcripts are highly lineage-specific or developmental stage-specific or both ^36^. These observations suggest that lncRNAs may play important roles in lymphoid cells. Indeed, the biological functions of several lncRNAs are characterized in these immune cells. For instance, Ranzani *et al*. identified an lncRNA, Linc-MAF-4, which is Th1 cell-specific and plays important roles in helper T-cell differentiation ^40^. Linc-MAF-4 can recruit chromatin modifiers LSD1 and EZH2 to the promoter to repress the expression of MAF, a Th2-associated transcription factor. Small interfering RNA (siRNA)-mediated knockdown of linc-MAF-4 increases the expression of MAF and skews T-cell differentiation toward the Th2 phenotype. This study reveals the functional importance of lncRNAs in T-cell differentiation *in vitro*. T cells play important roles in modulating immune response during viral and bacterial infection. Interestingly, lncRNAs seem to have regulatory functions in these processes. For example, the lncRNA nettoie Salmonella pas Theiler’s (NeST) is specifically expressed in the mouse Th1 helper T cells. During viral and bacterial infections, this NeST regulates the expression of the cytokine interferon-gamma, thereby modulating the immune response mediated by the Th1 helper T cells ^37, 38^. When lncRNA expression in various helper T-cell subsets was profiled, a Th2-specific lncRNA named LincR-Ccr2-5'AS was identified and characterized ^41^. Knockdown of this lncRNA by shRNA compromises the ability of the cells to migrate to the lungs *in vivo*. Interestingly, the global gene expression changes of LincR-Ccr2-5'AS knockdown are similar to those of Gata3 depletion, suggesting that there may be a functional link between the lncRNA and the Gata3 transcription factor during T-cell differentiation and immune function.

In addition to finding T cells, Wang *et al*. found an lncRNA, lnc-DC, which seems to be exclusively expressed in human DCs ^42^. This lncRNA is required for optimal DC differentiation. Functionally, this transcript is involved in regulating DC-mediated T-cell activation, and mechanistically, it achieves this by direct binding to the signal transducer and activator of transcription 3 (STAT3) in the cytoplasm, which promotes STAT3 phosphorylation on tyrosine-705, thereby facilitating STAT3 activation. Collectively, these observations indicate that lncRNAs are involved in both lymphoid cell differentiation and modulating lymphoid cell-mediated immune responses.

## Future directions

Thanks to the widely used high-throughput transcriptomic profiling techniques, such as RNA-seq, the number of annotated lncRNAs exploded over the past few years. Loss-of-function and gain-of-function studies have revealed and likely will continue to reveal the regulatory roles of lncRNAs in hematopoiesis and other biological processes. Here, we discuss from our own perspectives some of the outstanding challenges and opportunities for future studies of lncRNA in hematopoiesis.

### Coding versus noncoding

Most lncRNAs were identified by computational analysis on transcriptomic datasets. These transcripts are annotated as noncoding by bioinformatics approaches. Though powerful, these computational algorithms only predict, but by no means demonstrate, the absence of coding ability/potential of those ORFs present on lncRNAs. Interestingly, however, recent observations indicate that some of the ORFs on lncRNAs are used by the translational apparatus, ribosome, to generate small peptides (reviewed in 43). Critically, the small peptides resulting from annotated lncRNAs can be biologically functional ^44– 46^. Thus, an outstanding question is how many lncRNAs are truly noncoding and how many of these computationally annotated lncRNAs are actually coding transcripts? One approach to address this question is to test the RNA and ribosome association by using polysome analysis for specific transcripts or ribosome profiling for the whole transcriptome ^47^. Discriminating coding versus noncoding of the computationally annotated lncRNAs will provide important insights into whether the function of the transcript is mediated by the RNA itself or the polypeptides it encodes.

### Testing long noncoding RNA functions *in vivo*

Studies from hematopoietic cell lines and cultured primary cells revealed the regulatory roles of several lncRNAs in hematopoiesis. Whether the phenotypes from these loss-of-function studies *in vitro* can be recapitulated *in vivo*, however, is unknown. Thus, it is important to use knockout mouse models to determine the *in vivo* functions of lncRNAs. In addition, knockout mouse models can solve some technical challenges of *in vitro* lncRNA studies. For example, some lncRNAs, particularly those localized in the nucleus, cannot be efficiently knocked down by current shRNA/siRNA methods. Therefore, absence of phenotypes in these loss-of-function studies is uninterpretable. In addition, shRNA/siRNA approaches may have off-target effects. Thus, complete depletion of lncRNA locus using a mouse model can provide unambiguous information for establishing roles of lncRNAs in hematopoiesis.

Importantly, several lncRNA knockout mouse models have been generated to address this important question. Encouragingly, knocking out Xist in female mice, which comprises maintenance of X-inactivation in females, severely inhibits HSC maturation and results in highly aggressive myeloproliferative neoplasm and myelodysplastic syndrome with full penetrance ^48^. Similarly, knocking out Dlue2, an lncRNA frequently deleted in lymphocytic leukemia 2, in mouse results in dys-regulation of cell cycle progression and apoptosis of B cells. Interestingly, the mouse develops a chronic lymphocytic leukemia (CLL) that is similar to human CLL. These two lncRNA *in vivo* studies clearly indicate that proper expression of lncRNA is required for normal hematopoiesis. An important issue associated with the lncRNA mouse model is to discriminate whether the phenotype is caused by the RNA or by the DNA sequence that can potentially function as regulatory cis-elements. Bassett *et al*. provided an insightful discussion of this caveat ^49^. The rapid development and application of targeted genome editing technologies, such as the CRISPR-Cas9 system, greatly facilitate the generation of targeted gene-altered mouse models. We believe that these technical advances will be of great help in functional characterizing lncRNAs *in vivo*.

### Molecular mechanisms of long noncoding RNA-mediated regulation of gene expression

LncRNA can regulate gene expression via diverse mechanisms ^10– 14^. One common theme of lncRNA-mediated regulation of gene expression is that lncRNA recruits protein partners to exert its biological effect(s). Although not all protein-binding events on lncRNA will result in functional consequence ^50^, identifying the protein(s) that lncRNAs specifically associated with is an important first step to characterize the molecular mechanisms. To this end, an expanding number of biochemical methods for RNA purification coupled with mass spectrometry have been established ^51– 53^. Usually, many proteins that specifically bind target lncRNA can be identified from the mass spectrometry analysis. The next important question is to test the functional importance of the identified RNA-protein interactions. This can be achieved by structure-function mapping to reveal critical regions on the RNA that are important for recruiting the protein partner(s), and then mutant lncRNA can be generated to test the functional consequence of disrupting the RNA-protein interaction. One caveat of the biochemical approaches is that they usually require a large amount of material. This can be challenging for some lowly expressed lncRNAs. Thus, one complementary approach is to use single-molecular RNA fluorescent *in situ* hybridization to directly visualize lncRNA localization within the cell. This can provide important insights into the molecular mechanisms of lncRNA. For instance, this cell biology approach can determine whether the target lncRNA is localized to functional subcellular compartments, such as paraspeckles that are involved in splicing, certain chromatin regions, and co-localization with candidate proteins. In combination, the biochemical approaches and cell biology approaches can greatly facilitate the characterization of molecular mechanisms of lncRNA-mediated regulation of gene expression in hematopoiesis.

## Abbreviations

CLL, chronic lymphocytic leukemia; DC, dendritic cell; EGO, eosinophil granule ontogeny; HSC, hematopoietic stem cell; lncRNA, long noncoding RNA; MEP, megakaryocyte-erythroid progenitor; NeST, nettoie Salmonella pas Theiler’s; ORF, open reading frame; RNA-seq, RNA sequencing; shRNA, short hairpin RNA; siRNA, small interfering RNA; STAT3, signal transducer and activator of transcription 3.

## References

[1] Derrien T Johnson R Bussotti G: The GENCODE v7 catalog of human long noncoding RNAs: analysis of their gene structure, evolution, and expression. *Genome Res.* 2012;22(9):1775–89. 10.1101/gr.132159.111 22955988 PMC3431493

[2] Cabili MN Trapnell C Goff L: Integrative annotation of human large intergenic noncoding RNAs reveals global properties and specific subclasses. *Genes Dev.* 2011;25(18):1915–27. 10.1101/gad.17446611 21890647 PMC3185964

[3] Flynn RA Chang HY: Long noncoding RNAs in cell-fate programming and reprogramming. *Cell Stem Cell.* 2014;14(6):752–61. 10.1016/j.stem.2014.05.014 24905165 PMC4120821

[4] Batista PJ Chang HY: Long noncoding RNAs: cellular address codes in development and disease. *Cell.* 2013;152(6):1298–307. 10.1016/j.cell.2013.02.012 23498938 PMC3651923

[5] Kapranov P Cawley SE Drenkow J: Large-scale transcriptional activity in chromosomes 21 and 22. *Science.* 2002;296(5569):916–9. 10.1126/science.1068597 11988577

[6] Ponjavic J Ponting CP Lunter G: Functionality or transcriptional noise? Evidence for selection within long noncoding RNAs. *Genome Res.* 2007;17(5):556–65. 10.1101/gr.6036807 17387145 PMC1855172

[7] Guttman M Donaghey J Carey BW: lincRNAs act in the circuitry controlling pluripotency and differentiation. *Nature.* 2011;477(7364):295–300. 10.1038/nature10398 21874018 PMC3175327

[8] Harries LW: Long non-coding RNAs and human disease. *Biochem Soc Trans.* 2012;40(4):902–6. 10.1042/BST20120020 22817756

[9] Esteller M: Non-coding RNAs in human disease. *Nat Rev Genet.* 2011;12(12):861–74. 10.1038/nrg3074 22094949

[10] Wang KC Chang HY: Molecular mechanisms of long noncoding RNAs. *Mol Cell.* 2011;43(6):904–14. 10.1016/j.molcel.2011.08.018 21925379 PMC3199020

[11] Lee JT: Epigenetic regulation by long noncoding RNAs. *Science.* 2012;338(6113):1435–9. 10.1126/science.1231776 23239728

[12] Rinn JL Chang HY: Genome regulation by long noncoding RNAs. *Annu Rev Biochem.* 2012;81:145–66. 10.1146/annurev-biochem-051410-092902 22663078 PMC3858397

[13] Geisler S Coller J: RNA in unexpected places: long non-coding RNA functions in diverse cellular contexts. *Nat Rev Mol Cell Biol.* 2013;14(11):699–712. 10.1038/nrm3679 24105322 PMC4852478

[14] Quinn JJ Chang HY: Unique features of long non-coding RNA biogenesis and function. *Nat Rev Genet.* 2016;17(1):47–62. 10.1038/nrg.2015.10 26666209

[15] Satpathy AT Chang HY: Long noncoding RNA in hematopoiesis and immunity. *Immunity.* 2015;42(5):792–804. 10.1016/j.immuni.2015.05.004 25992856

[16] Nobili L Lionetti M Neri A: Long non-coding RNAs in normal and malignant hematopoiesis. *Oncotarget.* 2016. 10.18632/oncotarget.9308 27177333 PMC5226612

[17] Kondo M Wagers AJ Manz MG: Biology of hematopoietic stem cells and progenitors: implications for clinical application. *Annu Rev Immunol.* 2003;21:759–806. 10.1146/annurev.immunol.21.120601.141007 12615892

[18] Bryder D Rossi DJ Weissman IL: Hematopoietic stem cells: the paradigmatic tissue-specific stem cell. *Am J Pathol.* 2006;169(2):338–46. 10.2353/ajpath.2006.060312 16877336 PMC1698791

[19] Akashi K Traver D Miyamoto T: A clonogenic common myeloid progenitor that gives rise to all myeloid lineages. *Nature.* 2000;404(6774):193–7. 10.1038/35004599 10724173

[20] Traver D Miyamoto T Christensen J: Fetal liver myelopoiesis occurs through distinct, prospectively isolatable progenitor subsets. *Blood.* 2001;98(3):627–35. 10.1182/blood.V98.3.627 11468160

[21] Venkatraman A He XC Thorvaldsen JL: Maternal imprinting at the *H19-Igf2* locus maintains adult haematopoietic stem cell quiescence. *Nature.* 2013;500(7462):345–9. 10.1038/nature12303 23863936 PMC3896866

[22] Keniry A Oxley D Monnier P: The *H19* lincRNA is a developmental reservoir of miR-675 that suppresses growth and *Igf1r*. *Nat Cell Biol.* 2012;14(7):659–65. 10.1038/ncb2521 22684254 PMC3389517

[23] Luo M Jeong M Sun D: Long non-coding RNAs control hematopoietic stem cell function. *Cell Stem Cell.* 2015;16(4):426–38. 10.1016/j.stem.2015.02.002 25772072 PMC4388783

[24] Alvarez-Dominguez JR Hu W Yuan B: Global discovery of erythroid long noncoding RNAs reveals novel regulators of red cell maturation. *Blood.* 2014;123(4):570–81. 10.1182/blood-2013-10-530683 24200680 PMC3901070

[25] Paralkar VR Mishra T Luan J: Lineage and species-specific long noncoding RNAs during erythro-megakaryocytic development. *Blood.* 2014;123(12):1927–37. 10.1182/blood-2013-12-544494 24497530 PMC3962165

[26] Hu W Yuan B Flygare J: Long noncoding RNA-mediated anti-apoptotic activity in murine erythroid terminal differentiation. *Genes Dev.* 2011;25(24):2573–8. 10.1101/gad.178780.111 22155924 PMC3248679

[27] An X Schulz VP Li J: Global transcriptome analyses of human and murine terminal erythroid differentiation. *Blood.* 2014;123(22):3466–77. 10.1182/blood-2014-01-548305 24637361 PMC4041167

[28] Shi L Lin YH Sierant MC: Developmental transcriptome analysis of human erythropoiesis. *Hum Mol Genet.* 2014;23(17):4528–42. 10.1093/hmg/ddu167 24781209 PMC4119405

[29] Zhang X Lian Z Padden C: A myelopoiesis-associated regulatory intergenic noncoding RNA transcript within the human HOXA cluster. *Blood.* 2009;113(11):2526–34. 10.1182/blood-2008-06-162164 19144990 PMC2656274

[30] Iacovino M Hernandez C Xu Z: A conserved role for Hox paralog group 4 in regulation of hematopoietic progenitors. *Stem Cells Dev.* 2009;18(5):783–92. 10.1089/scd.2008.0227 18808325 PMC2775089

[31] Zhang X Weissman SM Newburger PE: Long intergenic non-coding RNA HOTAIRM1 regulates cell cycle progression during myeloid maturation in NB4 human promyelocytic leukemia cells. *RNA Biol.* 2014;11(6):777–87. 10.4161/rna.28828 24824789 PMC4156508

[32] Wagner LA Christensen CJ Dunn DM: *EGO*, a novel, noncoding RNA gene, regulates eosinophil granule protein transcript expression. *Blood.* 2007;109(12):5191–8. 10.1182/blood-2006-06-027987 17351112 PMC1890841

[33] Guttman M Amit I Garber M: Chromatin signature reveals over a thousand highly conserved large non-coding RNAs in mammals. *Nature.* 2009;458(7235):223–7. 10.1038/nature07672 19182780 PMC2754849

[34] Carpenter S Aiello D Atianand MK: A long noncoding RNA mediates both activation and repression of immune response genes. *Science.* 2013;341(6147):789–92. 10.1126/science.1240925 23907535 PMC4376668

[35] Pang KC Dinger ME Mercer TR: Genome-wide identification of long noncoding RNAs in CD8 ^+^ T cells. *J Immunol.* 2009;182(12):7738–48. 10.4049/jimmunol.0900603 19494298

[36] Casero D Sandoval S Seet CS: Long non-coding RNA profiling of human lymphoid progenitor cells reveals transcriptional divergence of B cell and T cell lineages. *Nat Immunol.* 2015;16(12):1282–91. 10.1038/ni.3299 26502406 PMC4653072

[37] Collier SP Collins PL Williams CL: Cutting edge: influence of *Tmevpg1*, a long intergenic noncoding RNA, on the expression of *Ifng* by Th1 cells. *J Immunol.* 2012;189(5):2084–8. 10.4049/jimmunol.1200774 22851706 PMC3424368

[38] Gomez JA Wapinski OL Yang YW: The NeST long ncRNA controls microbial susceptibility and epigenetic activation of the interferon-γ locus. *Cell.* 2013;152(4):743–54. 10.1016/j.cell.2013.01.015 23415224 PMC3577098

[39] Petri A Dybkaer K Bøgsted M: Long Noncoding RNA Expression during Human B-Cell Development. *PLoS One.* 2015;10(9):e0138236. 10.1371/journal.pone.0138236 26394393 PMC4578992

[40] Ranzani V Rossetti G Panzeri I: The long intergenic noncoding RNA landscape of human lymphocytes highlights the regulation of T cell differentiation by linc-MAF-4. *Nat Immunol.* 2015;16(3):318–25. 10.1038/ni.3093 25621826 PMC4333215

[41] Hu G Tang Q Sharma S: Expression and regulation of intergenic long noncoding RNAs during T cell development and differentiation. *Nat Immunol.* 2013;14(11):1190–8. 10.1038/ni.2712 24056746 PMC3805781

[42] Wang P Xue Y Han Y: The STAT3-binding long noncoding RNA lnc-DC controls human dendritic cell differentiation. *Science.* 2014;344(6181):310–3. 10.1126/science.1251456 24744378

[43] Andrews SJ Rothnagel JA: Emerging evidence for functional peptides encoded by short open reading frames. *Nat Rev Genet.* 2014;15(3):193–204. 10.1038/nrg3520 24514441

[44] Pauli A Norris ML Valen E: Toddler: an embryonic signal that promotes cell movement via Apelin receptors. *Science.* 2014;343(6172):1248636. 10.1126/science.1248636 24407481 PMC4107353

[45] Anderson DM Anderson KM Chang CL: A micropeptide encoded by a putative long noncoding RNA regulates muscle performance. *Cell.* 2015;160(4):595–606. 10.1016/j.cell.2015.01.009 25640239 PMC4356254

[46] Payre F Desplan C: Small peptides control heart activity. *Science.* 2016;351(6270):226–7. 10.1126/science.aad9873 26816363 PMC5150219

[47] Ingolia NT: Ribosome profiling: new views of translation, from single codons to genome scale. *Nat Rev Genet.* 2014;15(3):205–13. 10.1038/nrg3645 24468696

[48] Yildirim E Kirby JE Brown DE: Xist RNA is a potent suppressor of hematologic cancer in mice. *Cell.* 2013;152(4):727–42. 10.1016/j.cell.2013.01.034 23415223 PMC3875356

[49] Bassett AR Akhtar A Barlow DP: Considerations when investigating lncRNA function *in vivo*. *eLife.* 2014;3:e03058. 10.7554/eLife.03058 25124674 PMC4132285

[50] Davidovich C Wang X Cifuentes-Rojas C: Toward a consensus on the binding specificity and promiscuity of PRC2 for RNA. *Mol Cell.* 2015;57(3):552–8. 10.1016/j.molcel.2014.12.017 25601759 PMC4320675

[51] Chu C Zhang QC da Rocha ST: Systematic discovery of Xist RNA binding proteins. *Cell.* 2015;161(2):404–16. 10.1016/j.cell.2015.03.025 25843628 PMC4425988

[52] Minajigi A Froberg JE Wei C: Chromosomes. A comprehensive Xist interactome reveals cohesin repulsion and an RNA-directed chromosome conformation. *Science.* 2015;349(6245): pii: aab2276. 10.1126/science.aab2276 26089354 PMC4845908

[53] McHugh CA Chen CK Chow A: The *Xist* lncRNA interacts directly with SHARP to silence transcription through HDAC3. *Nature.* 2015;521(7551):232–6. 10.1038/nature14443 25915022 PMC4516396

